# Engineering Factor Xa Inhibitor with Multiple Platelet-Binding Sites Facilitates its Platelet Targeting

**DOI:** 10.1038/srep29895

**Published:** 2016-07-19

**Authors:** Yuanjun Zhu, Ruyi Li, Yuan Lin, Mengyang Shui, Xiaoyan Liu, Huan Chen, Yinye Wang

**Affiliations:** 1 Department of Molecular and Cellular Pharmacology, Peking University School of Pharmaceutical Sciences, Beijing, China; 2 State Key Laboratory of Bioactive Substances and Function of Natural Medicine, Institute of Materia Medica, Chinese Academy of Medical Sciences and Peking Union Medical College, Beijing, China

## Abstract

Targeted delivery of antithrombotic drugs centralizes the effects in the thrombosis site and reduces the hemorrhage side effects in uninjured vessels. We have recently reported that the platelet-targeting factor Xa (FXa) inhibitors, constructed by engineering one Arg-Gly-Asp (RGD) motif into Ancylostoma caninum anticoagulant peptide 5 (AcAP5), can reduce the risk of systemic bleeding than non-targeted AcAP5 in mouse arterial injury model. Increasing the number of platelet-binding sites of FXa inhibitors may facilitate their adhesion to activated platelets, and further lower the bleeding risks. For this purpose, we introduced three RGD motifs into AcAP5 to generate a variant NR4 containing three platelet-binding sites. NR4 reserved its inherent anti-FXa activity. Protein-protein docking showed that all three RGD motifs were capable of binding to platelet receptor α_IIb_β_3_. Molecular dynamics simulation demonstrated that NR4 has more opportunities to interact with α_IIb_β_3_ than single-RGD-containing NR3. Flow cytometry analysis and rat arterial thrombosis model further confirmed that NR4 possesses enhanced platelet targeting activity. Moreover, NR4-treated mice showed a trend toward less tail bleeding time than NR3-treated mice in carotid artery endothelium injury model. Therefore, our data suggest that engineering multiple binding sites in one recombinant protein is a useful tool to improve its platelet-targeting efficiency.

Although anticoagulant and antiplatelet agents serve as the main treatment for thrombosis, they often cause high systemic bleeding risk^1^,^2^,^3^. In some particular cases, this risk is markedly increased, such as percutaneous coronary intervention (PCI), which usually uses these two classes of drugs together^4^,^5^. Targeted delivery of antithrombotic drugs may centralize the effects in the injured vascular wall and reduce the bleeding risk^6^. Recently, we engineered novel activated-platelet-targeting Factor Xa (FXa) inhibitors by introducing an Arg-Gly-Asp (RGD) motif into different locations within a potent FXa inhibitor, ancylostoma caninum anticoagulant peptide 5 (AcAP5)^7^,^8^. These novel anticoagulants can specifically binding to platelet receptor α_IIb_β_3_, and further reduce bleeding risk in mouse arterial injury model comparing with native FXa inhibitors^7^.

In many cases, acute thrombosis needs immediate antithrombotic therapy^9^,^10^,^11^. Therefore, the sooner and the more targeted drugs centralized at the injured vascular sites, the less systemic bleeding risk. However, the recombinant proteins with targeting function may have fewer opportunities to interact with their targets, comparing to other targeting drugs encapsulated in various carriers (e.g. liposomes and nanoparticles). Because they have only one specific binding site, while other targeting drugs carry multiple binding sites^12^. Even if their binding affinities to targets may be similar to that of drug carriers, the binding kinetics may be different. In addition, different binding kinetics may affect the drug’s efficacy^13^,^14^. Several reports have shown that more binding sites on the carrier surface could facilitate the target binding^15^,^16^,^17^,^18^.

In the present study, we constructed AcAP5 variant NR4 containing three α_IIb_β_3_-binding sites (RGD motifs), and evaluated its FXa-inhibiting and platelet-binding abilities. And we found that engineering AcAP5 with multiple platelet-binding sites can improve its delivery to activated platelets, thus reduce the bleeding risks.

## Results

### Construction of AcAP5 variant NR4 containing three platelet-binding sites

We have previously constructed three platelet-targeting anti-FXa AcAP5 variants by fusing one RGD motif to the C-terminus (NR1) or N-terminus (NR2), or mutating the residues R_65_E_66_E_67_ to R_65_G_66_D_67_ (NR3)^7^. *In vitro* FXa activity assays showed that NR1 has reduced anti-FXa effect, and NR2 and NR3 have similar anti-FXa activities, comparing with native AcAP5. Moreover, NR3 showed more consistency in therapeutic efficacy^7^.

**Figure 1:**
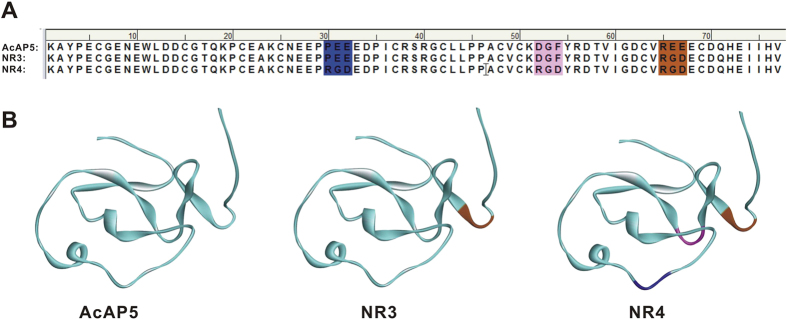
Protein sequences and the molecular models of RGD-containing AcAP5 variants. (**A**) The RGD-introducing sites in AcAP5. The mutated amino acids are shown in color background. (**B**) The model structure of AcAP5 and its variants. R_31_G_32_D_33_, R_52_G_53_D_54_ and R_65_G_66_D_67_ are colored blue, pink and brown, respectively.

Considering NR3 was the best AcAP5 variant, we appealed to generate a new AcAP5 variant NR4 containing three platelet-binding sites, using the similar strategy we constructed NR3^7^. A functional RGD motif needs to form a turn loop, which protrudes from the surface of protein structure, allowing its interaction with α_IIb_β_3_ receptor. Besides the site (R_65_E_66_E_67_) used in NR3, we found another two sites in AcAP5, P_31_E_32_E_33_ and D_52_G_53_F_54_, which may be suitable for introducing RGD motif by amino acids substitution. Both P_31_E_32_E_33_ and D_52_G_53_F_54_ are located on the surface of AcAP5, and their secondary structures are small loops. Therefore, neither amino acids mutation of P_31_E_32_E_33_ nor D_52_G_53_F_54_ to RGD would make a big change to the general structure of AcAP5 molecule. The NR4 variant was constructed by introducing three RGD motifs into AcAP5 molecule (Fig. 1A). The structure of NR4 was homology-modeled by MODELER program, and subjected to CHARMM energy minimization. The best model was selected and further verified using Profiles-3D and Ramachandran plot programs (Fig. 1B).

### NR4 retaining anti-FXa activity

**Figure 2:**
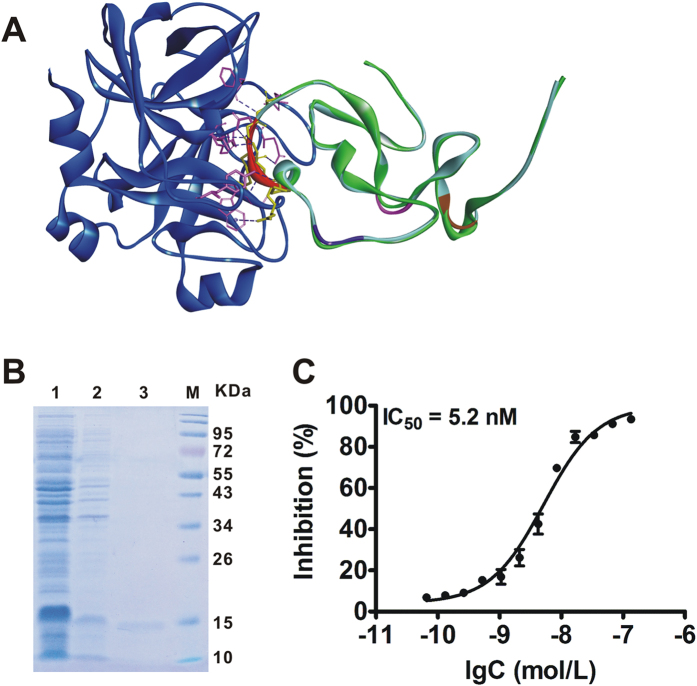
Molecular model of FXa-NR4 interaction and the FXa-inhibiting activity of NR4. (**A**) NR4 superimposed with AcAP5/FXa complex. FXa, AcAP5 and NR4 are in yellow, green and light cyan, respectively. The insertion-loops (R_38_S_39_R_40_G_41_) of AcAP5 and NR4 for binding FXa active site are highlighted in purple, and the RGD residues in NR4 are in blue, pink and brown ribbon, respectively. The interacting residues of FXa and NR4 are in cyan and purple stick models, respectively, while H-bonds are shown in dashed red lines. (**B**) Purification of NR4 using ion-exchange chromatography. Lane M, pre-stained protein marker; Lane 1, samples of cell lysates; Lane 2, 3, *protein* fractions eluted from anion- (Q sepharose) and cation- (SP sepharose) exchange columns, respectively. (**C**) NR4 inhibited the catalytic activity of FXa. The relative FXa inhibition (%) is shown as means ± S.E.M. from three independent experiments.

Since the introduced RGD motifs in NR4 are located away from the interaction interface between the insertion-loop (R_38_S_39_R_40_G_41_) and FXa active site, we suppose that their interaction would not be affected. As shown in Fig. 2A, the backbone of NR4 superimposed well with AcAP5, indicating that both proteins adopted a quite similar conformation. The molecular docking analysis revealed that the insertion-loop of NR4 formed strong intermolecular hydrogen bonds (H-bonds) with the active site of FXa, exactly the same with the binding mode between AcAP5 with FXa, indicating that its FXa-inhibiting activity may remain unaffected. Indeed, our data showed that NR4 inhibited the catalytic function of FXa at IC_50_ of 5.2 nM *in vitro* (Fig. 2C), which is equivalent to its parent AcAP5^7^. These data suggested that introduction of three RGD motifs in AcAP5 (NR4) did not affect its inherent anti-FXa activity.

### Computational analysis of the binding of NR4 with platelet α_IIb_β_3_ receptor

**Figure 3:**
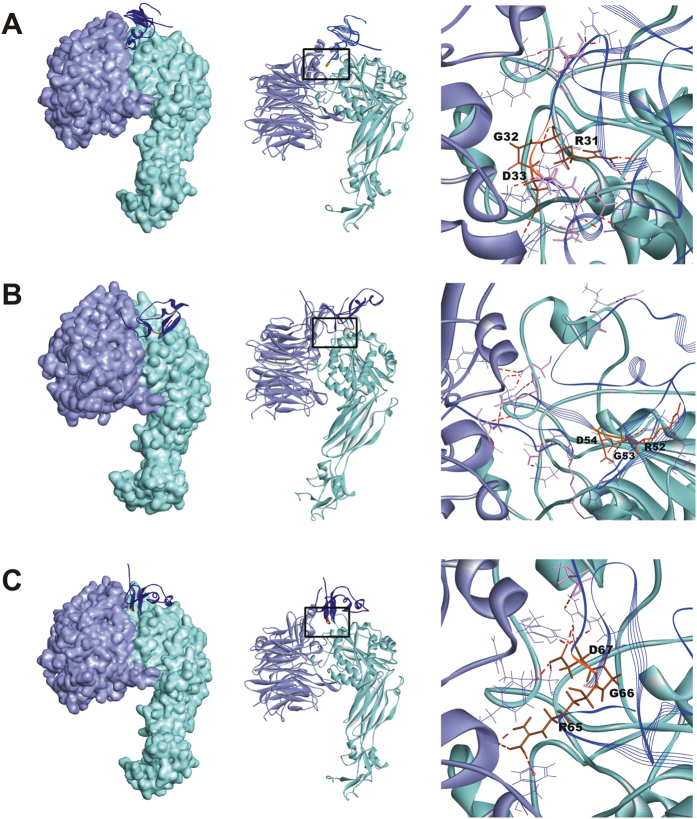
Protein-protein docking analysis of NR4-α_IIb_β_3_ interaction. Interaction surface between α_IIb_β_3_ receptor with each binding site of NR4, R_31_G_32_D_33_ (**A**), R_52_G_53_D_54_ (**B**) and R_65_G_66_D_67_ (**C**), respectively. Protein structure is shown as molecular surface model and/or ribbon model. The α_IIb_ and β_3_ subunits and NR4 are shown in light purple, light cyan, and blue, respectively. Intermolecular H-bonds between each binding site and α_IIb_β_3_ are shown in higher magnification (right panel). The interacting residues of α_IIb_β_3_ and NR4 are shown in lines and sticks, respectively, and the H-bonds are shown in dashed line (red).

To determine whether the introduced three RGD motifs in NR4 could interact with platelet α_IIb_β_3_ receptor, NR4 and α_IIb_β_3_ were subjected to molecular docking analysis. These RGD motifs are exposed on the surface of the AcAP5 molecule, allowing a favorable steric conformation to dock with the active pocket of α_IIb_β_3_ receptor. RGD residues could interact with α_IIb_β_3_ amino acids, forming several intermolecular H-bonds and salt-bridges (Fig. 3). In addition, some residues flanking RGD also favored RGD-α_IIb_β_3_ interaction. The non-bonded interaction energy was −126.3, −83.7 and −118.8 kcal/mol between α_IIb_β_3_ receptor with NR4_R31G32D33_, NR4_R52G53D54_, and NR4_R65G66D67_, respectively. R_65_G_66_D_67_ and R_31_G_32_D_33_ were more energetically favorable to interact with α_IIb_β_3_ than R_52_G_53_D_54_. Therefore, NR4 could adhere to activated platelets through any of the three RGD-α_IIb_β_3_ interaction modes.

### Multiple binding sites facilitating platelet-targeting delivery

**Figure 4:**
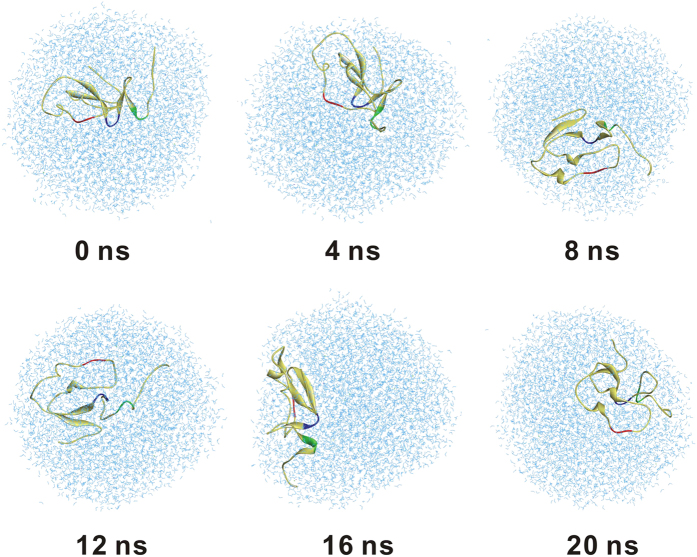
Molecular dynamics simulation of the physical movement of NR4 in periodic boundary conditions. The representative dynamics trajectory of NR4 in 20 ns MD simulations is displayed every 4 ns. NR4 is shown in yellow ribbon model, and its R_31_G_32_D_33_, R_52_G_53_D_54_ and R_65_G_66_D_67_ residues are colored red, blue and green, respectively. Water molecules wrapping around NR4 are shown as light blue sticks.

To test whether multiple binding sites of recombinant RGD-containing NR4 could promote its targeting to platelet, we performed molecular dynamics (MD) simulation to investigate the movement of NR4 physiologically. We assume that the ratio of ligand-receptor (NR4-α_IIb_β_3_) binding is always 1:1. So the probabilities of actual NR4-platelet binding can be reflected indirectly by the number of RGD-α_IIb_β_3_ occurrences. As shown in 20 ns MD trajectories, NR4 tend to move in a random manner (Fig. 4). Accordingly, it is unlikely to predict precisely how many times RGD motif of NR3 or NR4 appeared in a specific location. However, it is possible to tell which AcAP5 recombinant, NR3 or NR4, has more chances to bind with platelet. Our data in Fig. 4 showed that NR4 has more opportunities to interact with platelet α_IIb_β_3_ receptor in a specific timeframe, compared with NR3. Considering NR4 has 2 more platelet binding site than NR3, the MD results suggested that multiple binding sites in NR4 may facilitate platelet adhesion, leading to a better targeting effect.

**Figure 5:**
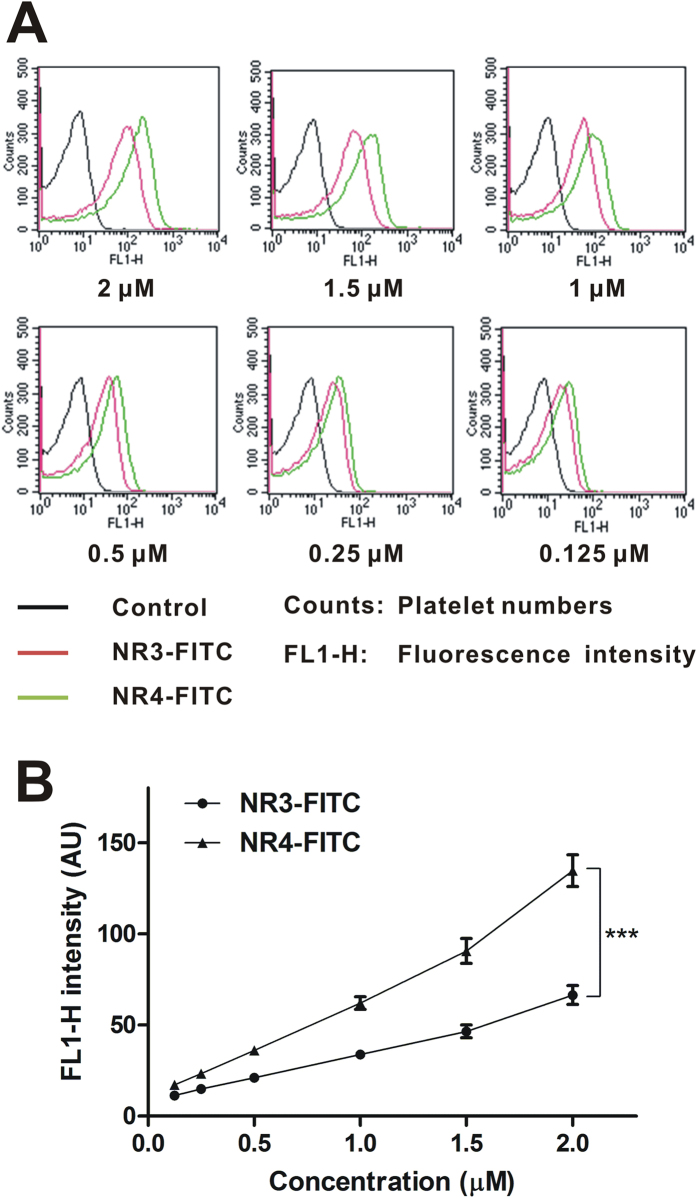
Flow cytometry analysis of NR3 and NR4 binding to platelet α_IIb_β_3_ receptor. (**A**) Representative histogram of flow cytometry showed that FITC-labeled NR3 and NR4 bind to activated platelet α_IIb_β_3_. (**B**) Statistical quantification of the mean fluorescence intensity of platelets showed the enhanced α_IIb_β_3_-binding for NR4-FITC. All data presented as means ± S.E.M. ***P < 0.001 by two-way ANOVA (n = 3).

To determine whether NR4 possess enhanced platelet targeting activity than NR3, an *ex vivo* flow cytometry assay was performed using protein N-terminal FITC-labeled NR3 and NR4. As shown in Fig. 5A, NR4-FITC exhibited a significantly increased binding to activated platelets compared to NR3-FITC, with an obvious right shift of the dose-response curve. With the increase of protein concentration (0.125, 0.25, 0.5, 1, 1.5 and 2 μM), the fluorescence intensity of platelets increased as well. Statistical quantification showed a significant enhanced platelet α_IIb_β_3_ binding for NR4-FITC than NR3-FITC at each concentration, respectively (Fig. 5B).

**Figure 6:**
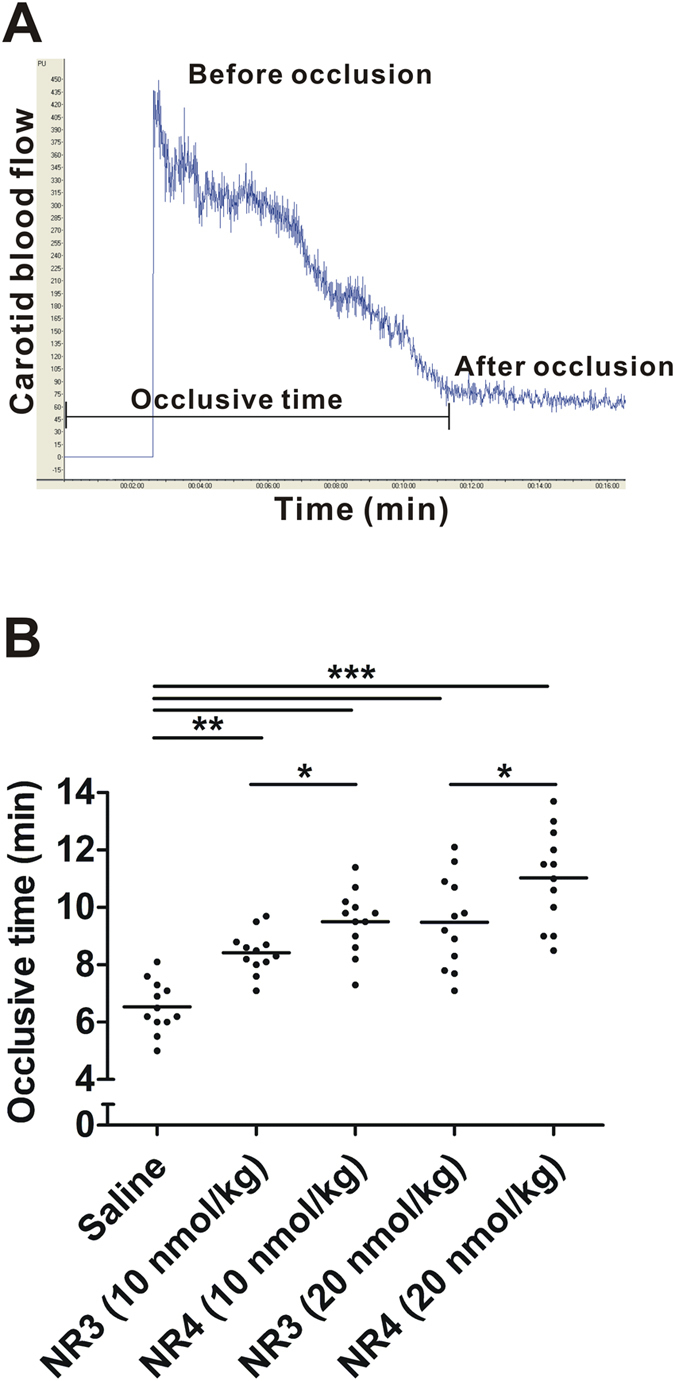
Prevention of carotid artery occlusion by NR3 and NR4 proteins. (**A**) Representative Doppler flow velocity of carotid arteries of rat for monitoring artery occlusion. (**B**) Recombinant NR3 and NR4 inhibit arterial thrombosis. Saline was injected as a negative control (0.5 ml/100 g body weight). Rats were treated with indicated doses of NR3 or NR4 (i.v.). The horizontal line indicates the mean occlusion time. Each symbol represents one animal. *P < 0.05, **P < 0.01, ***P < 0.001 by two-way ANOVA followed Bonferroni’s post hoc test; n = 12 each.

To further confirm that introduction of multiple binding sites to NR4 improve its adhesion to activated platelets *in vivo*, rats were subjected to an electrical-induction of carotid arterial thrombosis model in the presence of different concentrations of recombinant proteins. Both NR3 and NR4 prevented the platelet-rich thrombus formation dose-dependently as indicated by the significantly prolonged thrombosis occlusion time, suggesting that both RGD-containing variants could inhibit platelet aggregation (Fig. 6). Moreover, NR4 showed a clear and stronger inhibition on the thrombosis formation than NR3 at 10 and 20 nmol/kg, respectively. The two proteins have the same anti-FXa activity, and the only difference between them is that NR4 has two more RGD motifs than NR3. Therefore, the greater anti-platelet aggregation effects of NR4 might be attributed to its two additional RGD sequence. Since one NR3 or NR4 molecule could interact with only one platelet α_IIb_β_3_ receptor, these data collectively demonstrated that multiple RGD motifs promote platelet binding.

### Improved targeted delivery of antithrombotic drugs reducing bleeding risk

**Figure 7:**
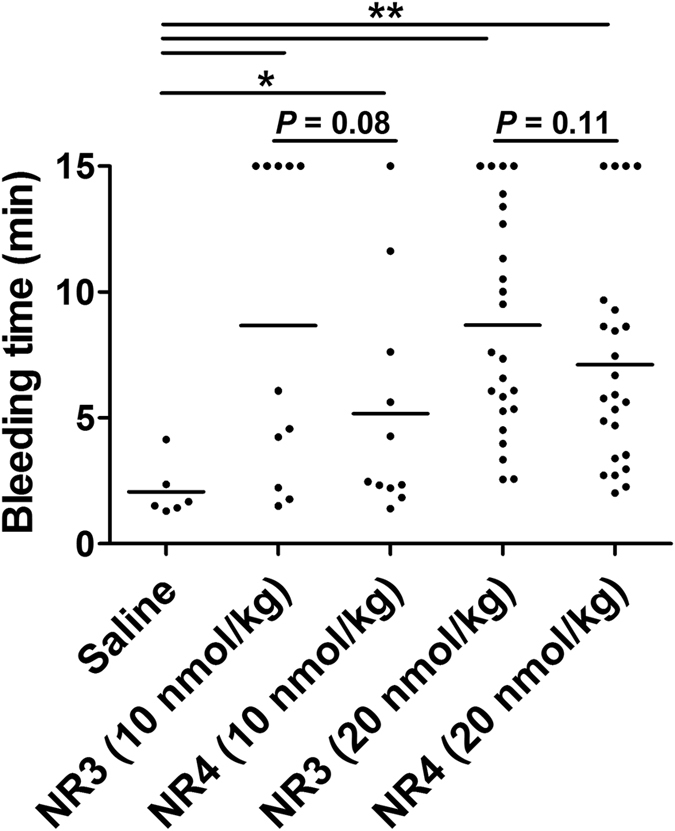
Bleeding time of NR3- or NR4-treated mice with arterial endothelial injury. Mice were injected i.v. with saline, NR3 or NR4 at 10 nmol/kg and 20 nmol/kg, respectively. Each dot represents the score for an individual mouse, and the horizontal line indicates the mean for each group. *P < 0.05, **P < 0.01 compared with saline group, by Mann-Whitney nonparametric test.

To determine whether improved platelet-targeted delivery of FXa inhibitors could further reduce the systemic bleeding risk in situations of vascular injury, mice were treated with 10% ferric chloride for 3 minutes to induce arterial endothelial injuries, and then subjected to a tail bleeding assay in the presence of different concentrations of recombinant proteins. We found that NR4-treated mice showed a trend of reduction in bleeding time compared with NR3-treated animals at a dosage of 10 nmol/kg (*P* = 0.08) and 20 nmol/kg (*P* = 0.11), respectively (Fig. 7). This result indicated that NR4 may concentrate more at the vascular endothelial injury site and mitigate the bleeding risks than NR3.

## Discussion

Ligand-receptor interactions are affected by high shear flow in the blood, which increases the difficulty of targeted delivery of the antithrombotic agents. To facilitate their targeted delivery, some antithrombotic agents can be delivered using biomimetic synthetic carriers such as liposome and nano-particles^12^. These drug carriers have multiple binding sites on the structure surface^12^. In contrast, antithrombotic proteins with targeting function usually contain only one binding-site. This implies that these proteins may have fewer opportunities to interact with targets, and may lead to a slower and/or less target binding.

Our recent works have shown that targeted delivery of the FXa inhibitors to activated platelets could centralize its anticoagulant effect at the injury sites and reduce the bleeding risk^7^. These platelet-targeted FXa inhibitors were constructed by introducing one RGD motif into an anticoagulant protein AcAP5, enabling additional competencies of targeted binding to platelet α_IIb_β_3_ receptor. Theoretically, the faster and the more these targeted anticoagulants adhered to activated platelets at the injured vascular sites, the better local anti-thrombotic effects achieved and the fewer systemic bleeding complication happened. To improve the targeted delivery, we wondered whether we could engineer multiple binding sites in one protein molecule, imitating drug carriers.

The target-binding unit in recombinant proteins can be large (e.g. an antibody or its fragment)^12^ or small (e.g. an affinity peptide)^7^. Because the binding unit is either fused to the N- or C- terminus of protein, or integrated within the protein sequence, it is difficult for a single protein molecule to have many functional binding units. Thus, recombinant proteins usually contain a single-binding-site, such as the RGD motif in NR3^7^. With the capability of interacting with platelet α_IIb_β_3_ receptor, the RGD motif has been served as a useful binding unit for targeting activated platelets^19^,^20^. As a functional RGD motif forms a small loop exposed on the protein surface^21^,^22^, we appealed to investigate whether engineering multiple RGD motifs in one protein could improve its platelet targeting. In this study, three sites on AcAP5, away from AcAP5-FXa binding interface^23^, were chosen for RGD motif introduction by amino acid mutagenesis, producing a recombinant AcAP5 variant NR4 containing three RGD motifs. Molecular superimposement indicated that NR4-FXa interaction was not affected. And FXa activity test confirmed that the FXa-inhibiting effect of NR4 remained unchanged. Protein-protein docking identified that all three introduced RGD motifs were able to anchor into the cavity between α_IIb_ and β_3_ subunits and form strong intermolecular H-bonds, suggesting that NR4 may interact with α_IIb_β_3_ receptor by anyone among these binding sites.

**Figure 8:**
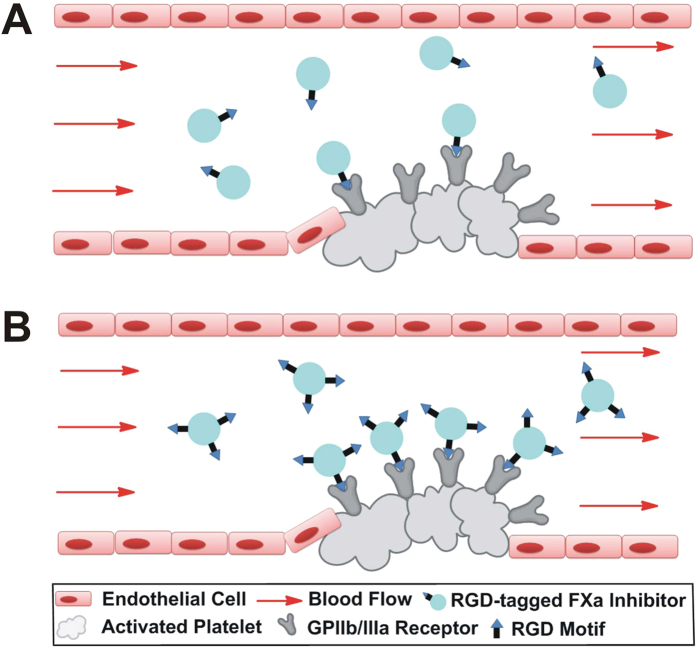
Proposed hypothesis of multiple binding sites improving the targeted delivery of RGD-tagged FXa inhibitors to activated platelets. Comparing with single-RGD-containing proteins (**A**), binding efficiency is higher in three-RGD-containing proteins (**B**), suggesting that platelet targeting efficiency is partially determined by the number of binding sites within the protein.

The binding of RGD-containing NR4 to activated platelets followed the basic principle of ligand-receptor interaction^24^, which ensured mutual recognition through the physically bumping or electrostatic interaction between ligand binding units (RGD motifs) and platelet binding epitopes (α_IIb_β_3_ receptor). Millions of NR4 hurtling at high speed in the bloodstream, their RGD motifs move occasionally close enough to platelet α_IIb_β_3_ receptor, and thus form stable binding between each other (Fig. 8). This is the initial stage of NR4-α_IIb_β_3_ binding for a successful targeted delivery of NR4 to activated platelets. The 20-ns MD simulated the movement of NR4 without constraints, and it showed that NR4 moved in a random mode. However, the probability of RGD motifs of NR4 appeared at an indicated location was higher than single-RGD-containing NR3, suggesting that NR4 may have more chances to interact with α_IIb_β_3_ receptor. Quantification of the binding between platelet and NR3/NR4 *ex vivo* using flow cytometry demonstrated an enhanced platelet targeting activity for NR4. Moreover, NR4 showed more potent antithrombotic effect in preventing arterial thrombosis than NR3 at both 10 and 20 nmol/kg. These data together indicated that multiple binding sites in NR4 can improve its platelet targeting, comparing with NR3 containing a single-binding-site. In addition, *in vivo* study showed a trend of less bleeding risk for NR4-treated mice in a model of carotid artery endothelium injury, compared with NR3-treated mice. Therefore, NR4 centralized more at sites of vascular injury and showed an improved antithrombotic benefit/bleeding risk ratio.

In summary, we constructed a recombinant AcAP5 variant NR4 containing three RGD motifs. NR4 preserved anti-FXa activity and acquired extra capability of binding to platelet receptor α_IIb_β_3_. Moreover, NR4 had more opportunities to bind to α_IIb_β_3_ than NR3 with single RGD motif, which increased its delivery efficiency and reduced the bleeding risk in mouse model of arterial injury. Our study serves as an evidence for improving platelet targeting by engineering multiple binding sites in one antithrombotic protein.

## Methods

### Ethics Statement

The experimental designs and all procedures were in accordance with the guidelines for the Care and Use of Laboratory Animals approved by Beijing Committee on Animal Care and Use. The experimental protocols were approved by the Committee on the Ethics of Animal Experiments of the Peking University Health Science Center. Efforts were made to reduce the number of animals used and their suffering.

### Homology modeling and molecular docking between RGD-containing protein NR4 and α_IIb_β_3_ receptor

To determine whether introduction of three RGD motifs to AcAP5 changes its interaction with α_IIb_β_3_ receptor, we first built the homology model of NR4 using the program MODELLER^25^ in the software package of Discovery studio 4.1 (Accelrys, San Diego, USA). Model structures of NR4 were evaluated by the Ramachandran plot^26^ as well as Profiles-3D programs^27^. The best model was selected and docked to integrin α_IIb_β_3_ receptor (PDB ID: 3ZE2) using protein-protein docking program ZDOCK^28^. Angular step size of ligand orientation was set to 6, generating a total of 54,000 poses. Subsequently, these poses were filtered by the prescribed amino acids of interaction interface. The remaining poses were further analyzed using RDOCK method^29^ with CHARMM force field^30^. The best interaction model for NR4-α_IIb_β_3_ complex was selected according to the score and energy. The non-bonded interactions energy between NR4 and α_IIb_β_3_ was also calculated as the sum of van der Waals (Evdw) and electrostatic (Eele) interaction terms.

### Plasmids construction for recombinant proteins

NR3 expression vector was generated previously^7^. The NR4 cDNA was generated by overlap PCR cloning. The PCR product was inserted into *pEASY*-T1 vector by TA cloning. After digestion with *Nde*I and *Bam*HI, the DNA fragments was sub-cloned into pET-30a(+) plasmid resulting in the recombinant expression plasmid pET30a-NR4. The plasmid was verified by DNA sequencing.

### Expression and purification of recombinant proteins

The *E. coli* BL21(DE3) was used to express recombinant proteins. Bacteria were grown at 37 °C till its OD_600_ reached 0.8 in LB media containing 100 μg/ml kanamycin. The expression of recombinant proteins was induced by adding 0.1 mM isopropyl-β-D-thiogalactoside (IPTG) for 10 h at 28 °C. Bacteria were harvested and re-suspended in buffer A (0.05 mol/L NaAc, 0.1 mol/L NaCl, pH 5.8). After sonication and centrifugation at 13,000 rpm for 15 min at 4 °C, the supernatant was collected and filtered with 0.45 μm membranes. The crude protein sample was subjected to anion exchange resin Q column (GE Healthcare, Uppsala, Sweden), and then eluted with buffer B (0.05 mol/L NaAc, 0.4 mol/L NaCl, pH 5.8). The fractions containing target proteins were collected, dialyzed and loaded onto a cation exchange resin SP column (GE Healthcare, Uppsala, Sweden), and eluted with buffer containing 0.5 mol/L NaCl (0.05 mol/L sodium citrate, pH 3.0). Collected proteins were separated by 15% (wt/vol) SDS/PAGE followed by Coomassie blue staining, and the purity of each fraction was evaluated. The purified proteins were then pooled and concentrated using an Amicon Ultra-15 device (Millipore) with a 3-KDa membrane cutoff and spun at 3,000 × g for 30 min. Protein concentrations were then determined by the BCA method following the manufacturer’s protocol.

### FXa activity assay *in vitro*

To determine the biological activity of each protein, an improved *in vitro* FXa assay was performed as described previously^7^. The assay was conducted in a 96-well plate. Each well contained 50 μl HBSA (10 mM HEPES, 150 mM NaCl and 0.1% bovine serum albumin, pH 7.5), 50 μl test sample diluted in HBSA (or HBSA alone for control) and 50 μl FXa enzyme (final concentration of 0.5 nM; New England Biolabs, Hitchin, UK). Following 30 min incubation at 25 °C, 50 μl Spectrozyme FXa substrate (final concentration of 0.25 nM; American Diagonostica Inc., Stamford, USA) was added to the well with total volume of 200 μl. The optical density (OD) at 405 nm was measured by using a microplate reader (Thermo Fisher Scientific, Vantaa, Finland) at 0 min and 10 min, respectively. The FXa-inhibiting activity was calculated using the following formula: Inhibition % = (ΔOD^10^_con_ − ΔOD^10^_test_)/ΔOD^10^_con_ × 100%, wherein ΔOD^10^_con_ means the difference of OD_405_ at 0 min and 10 min without proteins, and ΔOD^10^_test_ means the difference of OD_405_ at 0 min and 10 min with objective proteins.

### Molecular dynamics (MD) simulation

MD simulations with explicit solvent were conducted with CHARMM program using the CHARMM36 all-atom force field. The initial structures were solvated in water box, and followed by energy minimization with first 10,000 steps of steepest descent and another 2,000 steps of conjugate gradient. Then the system was heated gently from 0 K up to 300 K in 500 ps, and equilibrated at 300 K for 500 ps. The production runs were performed for 20 ns trajectory analysis in NPT ensemble, with a time step of 2 fs enabled by the SHAKE algorithm. A snapshot of the current conformation was recorded every 2 ps. The time-curves of the temperature, total energy and the root mean square deviation (RMSD) of Cα atoms from initial structures were calculated using 20 ns MD trajectories to determine whether the structures were balanced.

### Flow cytometry

Recombinant NR3 and NR4 proteins were labeled with FITC at the N-terminus (Bioss Antibodies, Beijing, China). Rat blood was collected in a tube containing 3.8% sodium citrate. Platelet-rich plasma (PRP) was collected by centrifugation at 18α for 10 min, and washed twice by PBS. Then PRP was resuspended and diluted in Tyrode’s buffer (150 mmol/L NaCl, 2.5 mmol/L KCl, 12 mmol/L NaHCO_3_, 2 mmol/L MgCl_2_, 2 mmol/L CaCl_2_, 1 mg/mL BSA, 1 mg/ml dextrose; pH 7.4)^31^. Diluted PRP (2 × 10^8^ platelets/ml) was incubated with saline or FITC-labeled NR3 or NR4 for 10 min, and then activated with 20 μM ADP for 5 min at 37 °C. Subsequently, samples were fixed with 1% paraformaldehyde, and measured by FACSCalibur (BD Bioscience, San Jose, USA).

### Electrical injury model for Doppler flow velocity measurement of the carotid artery

An improved method for measuring rat carotid artery thrombosis time was established, combining electrical stimulation-induced arterial thrombosis formation and detection of the carotid blood flow by laser Doppler flowmetry. Rat carotid injury model was carried out as previously described^7^. Briefly, rats were anesthetized with chloral hydrate (350 mg/kg, i.p.), and the left common carotid artery was surgically exposed. Five minutes after administration of normal saline or recombinant proteins, an occlusive thrombus formation was induced by electrical stimulation with 5 mA current for 2 min. The laser Doppler flow-probe (PeriFlux System 5000, Perimed AB, Jarfalla, Sweden) was placed above the carotid artery post injury to investigate the variation of blood flow, which decreased to a steady value indicating thrombotic occlusion.

### Bleeding time

Bleeding time was performed as we previously described^7^. Briefly, male ICR mice (25–28 g) were anesthetized with chloral hydrate (500 mg/kg, i.p.). To induce vascular endothelial injuries, a small piece of filter paper (2 × 4 mm) saturated with ferric chloride solution (10%) was placed under the left common carotid artery for 3 minutes under a dissecting microscope^32^. Mice were injected intravenously through the sublingual vein with either Saline or recombinant proteins (10 or 20 nmol/kg) 1 minute before the injury. Three minutes later, the tail was transected 2 mm from the tip and immediately submersed into a tube with saline at 37 °C. The bleeding time was recorded when visible blood stream was ceased for 2 minutes. If the bleeding did not stop, observation was terminated after 15 minutes.

### Statistical analysis

All data are presented as means ± S.E.M. Statistical analyses were performed using the two-way ANOVA, Bonferroni’s post hoc test, or nonparametric Mann-Whitney test as indicated. *P* < 0.05 was considered statistically significant.

## Additional Information

**How to cite this article**: Zhu, Y. *et al*. Engineering Factor Xa Inhibitor with Multiple Platelet-Binding Sites Facilitates its Platelet Targeting. *Sci. Rep.* **6**, 29895; doi: 10.1038/srep29895 (2016).
